# Exploring key stakeholder perceptions on the impact of younger stroke: a multinational, qualitative interview study

**DOI:** 10.1136/bmjopen-2025-114949

**Published:** 2026-06-28

**Authors:** Oisín Cleary, Jenny Davison, Orla McBride, Niamh Kennedy

**Affiliations:** 1 School of Psychology, Ulster University, Coleraine, UK

**Keywords:** Stroke, QUALITATIVE RESEARCH, Quality of Life, Caregivers

## Abstract

**Abstract:**

**Objectives:**

To explore the impact of stroke on young survivors.

**Design:**

Qualitative study design using semistructured interviews that underwent thematic analysis.

**Setting:**

Participants were recruited globally, with the majority of data collection occurring in the UK. All participant interviews were conducted online.

**Participants:**

This study is the first to explore the poststroke impact of young stroke survivors across three key stakeholder groups: young stroke survivors (n=22), young stroke caregivers (n=5) and stroke healthcare professionals (n=9).

**Results:**

Following 36 participant interviews, four primary themes emerged: (1) relationships and psychosocial considerations; (2) disrupted occupations and financial worries; (3) out of sight, out of mind and (4) acknowledgement of current healthcare practices and their efficacy. These results extend the findings from existing young stroke literature while providing multiple perspectives confirming that the impact is far-reaching, affecting caregivers, family and wider social circles.

**Conclusions:**

Participants reported a range of poststroke impacts specific to a younger cohort. These should be considered when developing and engaging in service provision for young stroke survivors to ensure an inclusive, person-led approach that goes beyond ‘one-size-fits-all’.

**STRENGTHS AND LIMITATIONS OF THIS STUDY**

- Multistakeholder approach.
- Interviews address less visible impacts of poststroke life.
- Recruitment of caregivers proved challenging.

## Introduction

 Stroke onset is highly impactful on an international scale, leading to a wide variety of unmet needs identified in existing stroke literature.^1^,^3^ Poststroke impact has been extensively documented in the stroke population across physical, cognitive, psychological, occupational, economic and social aspects of life and can affect the survivor themselves, their loved ones or wider society.^4 5^ These trends may be more complex to identify among young stroke (YS) survivors, typically defined as occurring among those aged between 18 and 65 years^4^, as recent evidence suggests younger cohorts may feature unique needs on account of their age.^6^,^8^ Some research has proposed that the impact of stroke may be more evident among YS survivors due to their current stage of life, in addition to them tending to live longer with their impairments, be of working age and have caring responsibilities for dependents.^9^,^11^ Despite following a similar stroke pathway to typically aged stroke survivors, YS survivors normally exhibit a higher lifetime economic burden in comparison.^12 13^

These observations are of particular concern as YS incidence and prevalence rates continue to increase,^14 15^ resulting in the average age of stroke onset to decline. Despite these trends, there remains minimal evidence capturing this impact among a YS population.^15^ Furthermore, qualitative stroke research typically features one participant group, that of stroke survivors^16^ or stroke caregivers,^17^ with few examples incorporating more than one stakeholder group^18 19^ and fewer still focusing on YS survivors.^6^ Gleaning evidence from multiple perspectives in stroke research allows for more diverse data to be collected to inform future healthcare practices, policies and research while simultaneously highlighting potential gaps/differences across stakeholder groups.^20 21^ This is especially important as YS onset can impact on the survivor, as well as their family and wider social circle.^22^ This study seeks to establish findings that compound on those raised in existing literature through its use of multiple perspectives. Accordingly, the following research study aims to explore and compare themes relating to the impact of younger stroke across three key stakeholder groups, in order to better inform future YS practice and research.

## Methodology

### Design

This study used a series of 1-on-1, semistructured interviews conducted online across three key stakeholder groups (see the ‘Participants and recruitment’ section) from November 2021 to June 2023. Informed consent was obtained from all participants prior to the commencement of the interview. Confidentiality of participant data was assured and securely stored.

### Participants and recruitment

This study features a multinational scope, exploring interview responses across three key stakeholder groups: YS survivors, YS caregivers and healthcare professionals (HCPs) including psychologists and physiotherapists, occupational and speech and language therapists with experience treating younger stroke. Each participant group had inclusion criteria: YS survivors were individuals with a clinically diagnosed ischaemic/haemorrhagic stroke that occurred between the ages 18–65 years of age. For the purposes of this study, YS survivors were included if they experienced their stroke between 18 and 65 years of age, regardless of their current age at the point of recruitment. Informal (ie, unpaid) caregivers of YS survivors, including spouses, family members or friends of the YS survivor who had an active role in their care were also included. Lastly, eligible HCPs required professional experience treating or aiding in the recovery of YS survivors. General and cohort-specific infographics were generated for all participants and their respective subcohorts. The recruitment strategy involved stroke charities contacting the three key stakeholders through their respective mailing lists. Similarly, recruitment took part in wider European, American and Australian mailing lists and social media platforms (eg, Stroke Alliance for Europe, American Stroke Association) as well as relevant Facebook stroke groups. All infographics contained contact information for researchers and study link. As recruitment was conducted through these means, there may be potential bias, as all YS survivors and caregivers would need to be digitally literate and engaging with stroke charities or social media groups to access recruitment information.

### Patient and public involvement

Stroke survivors, caregivers and HCPs were involved in the initial design of study for the development of some interview questions, but were not involved in the data analysis, reporting or dissemination of the research.

### Procedure

One-on-one interviews were conducted online on Microsoft Teams by the first author, following a semistructured approach for each stakeholder group. This format was chosen to accommodate differing participant schedules and time zones, as well as supporting any potential communication needs exhibited by YS survivors. One-on-one interviews also encouraged a safe environment for participants to discuss any sensitive topics regarding their stroke with no judgement. All participant interviews followed a consistent interview schedule for each stakeholder group (see online supplemental material 1), with variation emerging in participant responses. Once all assigned questions were issued, a debrief was communicated and the interview formally ended. Interview duration was a mean of 49.51 (SD=17.48) minutes. No incentives were offered for participation in the interview.

### Data analysis

All interview data was transcribed, pseudonymised and uploaded to NVivo V.15^23^ where it underwent thematic analysis. This process involved frequency-based systematic coding, establishing word patterns and the categorisation of relevant themes and subthemes^24 25^ by the first author. Codes were developed using short phrases that described the theme/subtheme being discussed by participants (eg, ‘occupational impact’). The second author also engaged in this process to ensure a rigorous approach, consistency between coding and intercoder reliability.^26^ Both authors identified themes separately across six participant interviews (two for each stakeholder group), then compared coding to ensure rigour, refinement and consensus of emerging themes. Interpretation of participant interviews falls on author reflexivity, and so it is important to highlight that the first author was an early career researcher new to qualitative research practices whereas the second author had several years more experience as a qualitative researcher. Though no conflicts arose during this process, the third author was ready to mediate this process. Interviewee quotes are demonstrated as their stakeholder group, participant number (ie, the last four digits of their phone number), the line number of the transcript from which the quote began (eg, S1234, L56) and their age. This paper has been reported in line with the Standards for Reporting Qualitative Research (SRQR) guidelines (see online supplemental material 3).^27^

## Results

### Participant demographics

36 participants were interviewed including 22 YS survivors, 5 YS caregivers and 9 HCPs (table 1). The majority (69.44%) of participants were recruited from the UK (n=25), with other international participation involving Canada (n=5), the USA (n=4), India (n=1) and South Africa (n=1).

**Table 1:** Participant demographics across three stakeholder groups

Group	YS survivors (n=22)	YS caregivers (n=5)	HCPs (n=9)
Sex–n (%)	16 female (72.73%), 6 male (27.27%)	2 female (40.00%), 3 male (60.00%)	6 female (66.67%), 3 male (33.33%)
Mean age (SD; range)	46.77 (10.13; 31–72)	55.20 (4.09; 51–62)	37.67 (9.64; 28–53)
Mean time in hospital, in weeks (SD; range)	6.95 (8.48; 1–32)	7.60 (7.77; 1–20)	N/A
Type of stroke–n (%)	10 ischaemic (45.45%), 11 haemorrhagic (50.00%), 1 unknown (4.55%)	N/A	N/A
Mean years of experience (SD; range)	N/A	4.84 (4.27; 0–10)	10.44 (8.65; 0–25)
HCP role	N/A	N/A	Occupational therapists (n=4), speech and language therapists (n=2), physiotherapist (n=1), doctor (n=1) and clinical psychologist (n=1)
HCP setting	N/A	N/A	Acute (n=5), community (n=4), early supported discharge (n=1), homecare (n=1)

HCP, healthcare professional; n, number of responses; N/A, not applicable; YS, young stroke.

### Thematic analysis results

Four themes emerged regarding the impact of younger stroke, including (1) relationships and psychosocial considerations; (2) disrupted occupations and financial worries; (3) out of sight, out of mind and (4) acknowledgement of current healthcare practices and their efficacy (table 2).

**Table 2:** Themes and the categorisation of evidence supporting them

Themes	Categorisation
Theme 1 – relationships and psychosocial considerations	1.1 – independent and community-driven quality of life 1.2 – personality, temperament and mood 1.3 – familial and romantic relationships
Theme 2 – disrupted occupations and financial worries	2.1 – interrupting progress on the career ladder 2.2 – the cost of young stroke
Theme 3 – out of sight, out of mind	3.1 – what is unseen? 3.2 – how others’ lives are impacted 3.3 – reflections on the good outcomes poststroke
Theme 4 – acknowledgement of current healthcare practices and their efficacy	4.1 – tolerance, motivation and neuroplasticity 4.2 – where current healthcare is failing 4.3 – what we can learn from good and bad practice

For information on which stakeholder group contributed to each theme, please see online supplemental material 2.

### Theme 1: relationships and psychosocial considerations

This theme describes many of the psychological and social consequences of stroke that can typically be found among younger individuals, as reported equally across stakeholder groups.

#### Category 1.1: independent and community-driven quality of life

Several aspects pertaining to quality of life emerged in participant interviews, most of which centred around activities of daily living (ADLs), loss of independence and inability to travel. Some ADLs were not unique to a younger cohort (eg, self-bathing, household tasks) but notably, ADLs relating to exercise and engaging in hobbies were among the highest reported. Many of these hobbies and exercises were ‘youthful’ and/or social (eg, tobogganing, music festivals/concerts and videogames) in nature, which in some cases negatively impacted on YS survivors’ social lives. Active socialisation exhibited mixed results, with some YS survivors finding it easier to make friends poststroke (usually with fellow survivors), but many accounted that a lot of existing friends drifted apart. Support groups were typically informative and beneficial in YS recovery, often leading survivors to volunteer at the support groups in question, resulting in a positive feedback loop. Despite this, there was often a lack of other YS survivors present at these support groups. This sometimes led to support and advice that was not beneficial to a young individual who had a stroke and was of working age. Unfortunately, many interview participants accounted that there were no support groups nearby, even on a national scale, leading many to engage in online supports such as through social media platforms and forums.

You need people to do absolutely everything for you, help to do absolutely everything for you. […] that total loss of independence (Survivor 0131, L172, age mid 40s).

#### Category 1.2: personality, temperament and mood

For YS survivors, health anxiety was widely discussed, especially the prospect of suffering yet another stroke or other cardiovascular condition. This trend was more apparent in individuals with an unknown aetiology, which can often be the case among younger cases of cryptogenic stroke.^28^ Unhappiness and depression were also referred to in young poststroke accounts. Some survivors with mental health concerns prestroke found their mental symptoms amplified poststroke. Similarly, self-harm, death ideation and suicidal ideation were experienced by some YS survivors. Changes in temperament/personality were also reportedly common among YS survivors, where they were quicker to temper, use foul language or more prone to say things they did not mean.

It’s kind of out before I’ve even thought of it (spoken word), and then I don’t necessarily think what I’ve just said either. So, it’s sort of an emotional outburst can happen. Sometimes unkind (Survivor 8523, L281, age mid 40s).

Similarly, some YS caregivers accounted that the survivor under their care exhibited a change in their personality following their stroke. This personality shift was occasionally compared in likeness to that of a child, which when exhibited in a fully grown adult following brain injury, could be quite distressing for many caregivers in this area.

I thought, I’m coming home to a toddler, you know, like just someone… I’m just minding someone who will only do what’s suggested to them, you know, I’m not actually living with another adult (Caregiver 7863, L218, age mid 50s).

All of these factors made it difficult for some YS survivors to reintegrate into society, especially during a more social chapter in their life.

#### Category 1.3: familial and romantic relationships

Interview participants detailed that a YS survivor’s family was integral and often supportive in their recovery, but it is common that they were unsupported themselves, especially with regards to mental health and financial issues. This was of particular concern to YS survivors who had children, frequently highlighting the potential trauma they, and any other family member, may feel poststroke, especially in younger individuals where stroke was often never considered a possibility. Also raised was the fact that many participants were of childbearing age and were unable to have children any more, with all stakeholder groups detailing how distressing this can be. Similarly, many YS survivors were primary caregivers prior to their stroke (either for children or elderly parents), and navigating a poststroke caring role, while sometimes requiring care themselves, was reportedly difficult to navigate.

I would lead us and like […] “let’s go for this hike, let’s build a snowman, let’s go tobogganing” and I was right there doing that with my children all the time. And I can’t do that anymore (Survivor 5453, L158, age early 50s).

Spousal and partner relationships were often more strained poststroke, occasionally ending in separation or divorce. All interviewed caregivers found that the relationship that they have with those under their care had changed poststroke, either positively or negatively. Notably, issues regarding intimacy and sexual activity were raised in a minority of YS survivors, but this was mentioned much more frequently among YS caregivers and HCPs.

### Theme 2: disrupted occupations and financial worries

This theme describes many of the occupational and financial consequences of stroke that can typically be found among younger individuals and their support network as reported across all participant groups.

#### Category 2.1: interrupting progress on the career ladder

Many YS survivors/caregivers are in full-time/part-time employment/education when a stroke occurs, leading many to cease or be forced to leave their role, due to an impairment or need of care affecting their performance. Accordingly, many HCPs commented that return to work was often a primary goal for YS survivors when rehabilitating some of their impairments. The aspects of stroke that affect job performance were widely varied. Despite this, the most commonly elicited impairments that contributed to this vocational disturbance were physical fatigue and mobility for those who work physically demanding jobs, and mental fatigue and impaired memory, attention and executive functioning for those who work cognitively demanding jobs.

You have a highly cognitive job. One of my clients had just gotten a job at a bank and um, now she has trouble with that, and she can’t go back to work (HCP 0782, L96, age early 30s).

Some survivors found themselves back at work/education after a period of recovery, but this was often at a part-time or flexible capacity with reduced overall hours per week (and subsequently reduced income if working). Positive work environments with aid from employers, colleagues and vocational therapy appeared to mitigate this impact, but many of these facets can be challenging, especially for self-employed individuals.

Biggest change has been that because of the stroke, I can’t work and because I can’t work, I lost the income (Survivor 7151, L115, age late 50s).

The occupational impact of younger stroke is not restricted to the survivors, as YS caregivers were shown to take on additional responsibilities for increased income or having to take reduced hours or leave their job in pursuit of their sudden caring role.

I was working full time before [YS survivor’s] injury and after their injury I… still maintained full time work, but in reality it was not sustainable (Caregiver 7863, L122, age mid 50s).

#### Category 2.2: the cost of young stroke

Often referred to in tandem with the occupational impact of younger stroke, many participants accounted on the topic of financial difficulties and worries that may occur. The majority of survivors identified a loss of income as one of the most impactful aspects of poststroke life. Various allowances may take place immediately poststroke such as sick/annual leave, but this was unavailable to many, especially those who were self-employed. Several survivors had to register for some form of financial support or ‘benefits’ system (eg, PIP in the UK) where complaints on the process were often raised.^29^

Not being able to earn and work, you know? I lost the earning years. The forties are supposed to be the highest earning years. I’m now 50 and I’m alone and struggling (Survivor 7703, L368, age early 50s).

In addition, a lot of UK-based survivors highlighted their decision to pay for their own treatment as they were dissatisfied with national healthcare wait times and efficacy, leading to yet more financial costs. Additional costs when making adjustments to living areas, early pensions/retirements, excessive sick pay, dipping into savings and the subsequent financial stress of all these areas were all demonstrated, causing a great need for increased financial aid poststroke amid younger individuals. Notably, many of these adjustments may already exist or not be necessary in typically aged stroke survivors or in older survivors within the 18–65 bracket (eg, those aged 55–65) who may already be retired or near retirement, providing an extra layer of financial assurance unavailable to younger survivors and caregivers, who may have more costs to consider.

More of my people who are older and have had strokes are more prepared to retire. Umm, my people, who are, you know, like say 27–32, are not ready to retire (HCP 0782, L66, age early 30s).

### Theme 3: out of sight, out of mind

This theme seeks to demonstrate the less observable aspects that many YS survivors and caregivers experience, as evidenced across both YS survivors and YS caregivers of this study.

#### Category 3.1: what is unseen?

At the start of the YS pathway, many survivors reported that a prompt diagnosis was not always achieved. Commonly suggested reasons were the lack of cardiovascular risk factors and presentation of ‘non-traditional’ symptoms of stroke, such as those demonstrated in the ‘Act F.A.S.T.’ public-health campaign.^30 31^

My stroke didn’t follow the F.A.S.T. rules. I had a headache and that was it (Survivor 2790, L180, age early 30s).

Interestingly, this conflicted with HCP reports, with many theorising that YS survivors express fewer comorbidities than their typically aged counterparts, meaning their stroke symptoms would be more readily self- or peer-recognised, leading to quicker diagnoses. Regardless, when asked about unseen outcomes poststroke, fatigue, visual impairment, certain language difficulties (more associated with symptoms of aphasia as opposed to dysarthria) as well as several cognitive, emotional, social and financial outcomes were raised. In some instances, young survivors themselves were unaware of their own impairments (especially cognitive impairments), having to be informed by other individuals, such as YS caregivers. Due to their unseen impairments, several YS survivors felt that the public treated them without special consideration, often feeling judged when performing ADLs (eg, paying at the till) more inefficiently than that of a healthy individual. In some instances, this contributed to several participants’ occupational (eg, job applications) and social impacts (eg, societal reintegration and social anxiety) following their stroke.

I’ve got a little lanyard that says that I’ve got a disability now, […] this lanyard helps make people aware that I’ve got speech problems (Survivor 7412, L178, age early 50s).

For some individuals, unseen impairments only became apparent when returning home, whereas it could be months or years before they were recognised or rehabilitated for others, if they were rehabilitated at all.

If a younger person comes into a hyperacute stroke unit with a mild stroke […] unseen difficulties can be missed, particularly maybe with the higher-level executive functions (HCP 3931, L369, age early 30s).

It commonly emerged that the general public had no way of knowing what YS survivors/caregivers go through supporting the false ideation that stroke is a ‘condition of the elderly.’ Many caregivers reported that those under their care appear physically healthy, which had positive connotations for some and difficult setbacks for others. With more awareness in the public, feelings of social anxiety and reluctance to reintegrate back into society would reportedly reduce and could also help mediate other impacts on YS survivor quality of living. Similarly, greater awareness from HCPs who are less familiar with treating YS could potentially aid in age-specific rehabilitation and in-hospital/post-discharge support.

There’s people (that) don’t drink, don’t smoke… they’re fit people that have had a stroke so… There’s more young people getting or having a stroke then you would ever imagine (Caregiver 1820, L456, age early 60s).

#### Category 3.2: how others’ lives are impacted

According to all stakeholders, primary caregivers often experience an overwhelming loss of independence, as well as difficulties associated with working in employment in addition to caring responsibilities. Similar to survivors, many YS caregivers are also responsible for the care of other individuals (such as young children or elderly parents). The time commitment one makes to pursue the care of not one, but several individuals could potentially compound on these aforementioned difficulties. Other aspects of YS caregivers’ lives that were raised as the most impactful included finances, social life, and in the overall change in relationship between the caregiver and survivor.

I don’t do an awful lot of stuff that isn’t directly or indirectly related to being a caregiver (Caregiver 2615, L60, age early 50s).

Frustration was common among YS caregivers of this study. In some instances, caregivers were angry at the survivor themselves for various reasons such as when support groups were not attended or interventions were not put into practice. Some caregivers got visibly upset during the interview when addressing such issues as they felt that they were dwelling in the past, becoming overwhelmed and experiencing difficulty in accepting events as they were. The onset of stroke is difficult for the individual, but the emotions exhibited by caregivers of YS survivors clearly show that the effects of stroke can have devastating impacts on surrounding individuals too. Many YS survivors and their caregivers had a mixture of both satisfactory and dissatisfactory support networks. Support from family and/or friends was excellent for some, likely aiding with the many stressors that can arise among YS survivors and their caregivers. Sadly, these support networks were inadequate at times, and instead of mitigating stressors, the lack of support was prone to exemplify them. As YS survivors are a subpopulation of the general stroke population, it was common for these survivors and their caregivers to desire the aid of support groups comprised of young people in similar situations to themselves. Additionally, many caregivers of YS survivors felt isolated as they knew very few individuals in a comparable situation to themselves. Conversely, others were able to find individuals who shared the unique insights one would acquire surviving or caring for a stroke at a young age.

I’ve struggled quite a bit. Um yeah, I would have liked a bit more help and guidance for me really. But there isn’t really. The focus for all the help goes on [YS survivor], which I understand ‘cause they’ve got physical needs as well, but. You know, um? I would have liked a bit more guidance and help (Caregiver 0095, L193, age mid 50s).

The majority of caregivers interviewed in this study felt abandoned by their respective healthcare service, especially in the later stages along the stroke recovery pathway. To combat this, it was many YS caregivers’ recommendation that they be provided with further information and guidance on what the exact stages of stroke recovery entail and improved general signposting to allow the seeking of any potential external aid that may benefit their journey. A minority of caregivers of YS survivors found that they had limited access to healthcare. Either there was no support in their area, or it was difficult to travel to an area where they could access the support they need. Additionally, there was little support regarding household adjustments or in the signposting of YS caregiver groups as reported by some individuals.

I haven’t received much additional support. But part of me doesn’t really know where I would look (Caregiver 7863, L39, age mid 50s).

#### Category 3.3: reflections on the good outcomes poststroke

Despite the life-altering event that YS survivors endured, several accounted a series of good outcomes as a result of their stroke. Young survivors often felt lucky to be alive, leading them to take on a different perspective on life. Life goals sometimes shifted, and in rare cases, survivors accounted how they would do it all again. Several participants, particularly those with high-stress jobs, confirmed that they had a more ‘laid back’ attitude and were happy with the prospect of taking a different career path. Having the experience of a stroke, some survivors accounted how they became more empathetic to others and made healthier life choices to minimise the risk of stroke reoccurrence. Many survivors had not thought of good impacts until it was posed as a question to think during the interview. It was common that, although initially there were no good impacts to report, with a little time and self-reflection, several good poststroke outcomes would be explored.

My stroke changed my life for the better. I wouldn’t undo it (Survivor 4434, L447, age early 70s [stroke onset in late 50s]).

Unfortunately, the majority of caregivers interviewed in this study had more difficulty expressing good outcomes poststroke. Some caregivers found that their lives were ‘going fast’ prestroke and that they could finally slow down. Additionally, several caregivers took pride in their ability to see and aid in the recovery of their spouse or family member following their stroke. Other good impacts specific to caregivers of YS survivors involved obtaining a greater awareness surrounding the area of stroke, the ability to help other caregivers in a similar situation, and general feelings of acceptance of the situation.

I’ll grab every moment of life and not get upset by things cause you know, once you go through something like this you realise it’s not worth being bothered by stuff (Caregiver 2615, L137, age early 50s).

### Theme 4: acknowledgement of current healthcare practices and their efficacy

This theme displays the current standard of care for YS survivors from HCPs in this area. Evidence for this theme was predominately informed by HCPs when referring to healthcare, but there are important points that support/refute this evidence from YS survivors/caregivers.

#### Category 4.1: tolerance, motivation and neuroplasticity

It was common for participants (especially HCPs) to compare YS survivors with more typically aged stroke survivors (ie, those aged >65) to showcase key differences in recovery, imbalances in care and the importance of one’s ‘stage in life’ when exploring outcomes poststroke. Some younger survivors may practise hobbies or exhibit behaviours stereotypically perceived to be more common in older individuals (eg, sedentary behaviour or an inactive social life), thus having less poststroke goals to reach and less motivation to do so. Motivation aside, bodily health, physical tolerance and fewer comorbidities may be common factors resulting in more favourable YS recovery, but these may not always be the case. These discussions gave way to a more complex, but important, distinction to be made in YS research. Several HCPs strongly believed that age shouldn’t be a determining factor for poststroke rehabilitation, but instead a consideration for the poststroke impact.

You’re going to inevitably have someone who’s 70 who would really benefit from that (younger) service but can’t access it or […] someone who’s 55 and there’s a really good older person service that would actually really suit them and they can’t access it. So, I just think anything based on age is probably, it’s not that helpful (HCP 1652, L566, age early 40s).

HCPs explored several factors that impact YS recovery. Goal setting, in particular the setting of age-specific goals, was found to have a positive impact, heightening motivation in YS survivors in pursuit of their recovery. A few core themes emerged in the younger cohort with regards to their recovery. First, many HCPs believed that their younger patients had higher levels of motivation and a greater tolerance to engage in longer and more intensive rehabilitative therapies, thus leading to better recovery.

My younger folks were like, “Yeah, I could keep improving forever!” (HCP 0782, L170, age early 30s).

Participants also described YS survivors as having a greater potential for recovery due to their age and better levels of neuroplasticity. Furthermore, they found YS survivors better at self-managing their own goals, both in-hospital and when returning home. Finally, several HCPs acknowledged that in a lot of cases, YS survivors typically have more to recover as their goals and expected standard of living poststroke tend to be set higher than that of their typically aged counterparts.

The expectations are different as well. People want to get back to what they were doing before (their stroke), and what they were doing before was at a higher level possibly (HCP 3931, L196, age early 30s).

#### Category 4.2: where current healthcare is failing

Many participants felt abandoned by their respective healthcare system, and this abandonment often took place once they were discharged from the hospital. This theme was universal across the various healthcare settings (nationalised such as through the NHS or insurance-based such as with USA healthcare), with issues relating to accessible rehabilitation potentially being exacerbated in insurance-based healthcare systems. With little exception, participants felt the need to self-advocate and to chase their own rehabilitation, as opposed to it being readily offered as part of their stroke recovery pathway.

When I went home my insurance coverage didn’t cover anything. […] It covered a little bit. I had 10 hours of at home therapy […] you know, 24/25 years ago. It seems a little lacking, right? (Survivor 8495, L84, age late 40s).

Many YS survivors highlighted that their rehabilitation brought them to a stage of recovery that they were dissatisfied with. Following rehabilitation, some YS survivors accounted that they were not treated like an individual, despite having an array of individualised needs and poststroke impacts. For most participants, they found that the information and rehabilitation they received was insufficient or irrelevant to their age group or life stage. Accounts on age-specific factors (eg, child support or youthful hobbies/activities) were rarely addressed in-person, with brochure handouts reportedly being uninformative or largely immaterial in this matter.

They just have this formula that they use. Um, they tell you all the time “every stroke is different,” but yet they’re treating you as a kind of template. It’s ridiculous! (Survivor 5907, L447, age mid 50s).

Regarding contact with healthcare staff, survivors expressed difficulty getting in contact with specialists that could aid in an aspect of their recovery once they were discharged. When considering that some poststroke impacts are completely unseen and take time to uncover, this raised an issue that some stroke symptoms remain untreated months or years after discharge, if at all. Several screening procedures were also raised, particularly cognitive screening in the acute setting. Many HCPs raised that cognitive screening conducted so early in the stroke pathway may not highlight all or any cognitive impairments found in YS survivors.

Some of our cognitive screens aren’t sensitive enough (HCP 5519, L32, age late 20s).

The remaining majority of YS survivors found that their healthcare needs were unmet to varying degrees. The most commonly reported unmet needs were lack of follow-up, the ‘one-size-fits-all’ approach and receiving information.

I was not treated as an individual in that respect at all, even when I asked to be. Life is a lot more than being able to get yourself to the bathroom and back, and to be able to make a simple meal for yourself… That’s not even OK for the typically aged stroke survivor (Survivor 5907, L275, age mid 50s).

#### Category 4.3: what we can learn from good and bad practice

Many HCPs believed that they were a part of a highly accessible service either locally or virtually. They also believed that the information they were personally providing to YS survivors was of a good quality, encompassing both signposting queries and adhering to age-specific frequently asked questions. Furthermore, many HCPs found themselves altering their standard rehabilitative practices (eg, zimmer frame use for mobility issues) in order to treat younger survivors to a more appropriate standard. These alterations were often inspired by young survivor goals. Accordingly, these goals were often determined by the different demands and milestones experienced by YS survivors. It was common for survivors to be engaging in activities reminiscent of their current life stage. Examples of these activities included driving, paying ongoing finances, maintaining an active social life and working in full-time employment, factors that can be apparent in older stages of life but often to a lesser extent. Relatability within support groups was a concern addressed by many HCPs, as YS survivors/caregivers were less likely to know of, or communicate with, other stroke survivors of a similar age, especially in males. Despite the information and signposting provided by HCPs, many YS survivors/caregivers still found that it was not enough, or in some cases would be too much. In a lot of cases (sometimes in reference to poor staffing/training), there was no signposting occurring. Almost all HCPs wished to carry out follow-ups on their stroke patients for as long as was needed but often could not due to their large caseloads. Despite detailing how they cater rehabilitative practices to younger survivors, many HCPs still acknowledged that the overall standard of practice is non-inclusive, again fulfilling that ‘one-size-fits-all’ mentality.

A lot of the timelines for specific stroke programs are geared around what would be reasonable for somebody who is older, right? And the reduced neural plasticity of someone who is older (HCP 0782, L174, age early 30s).

Some HCPs highlighted a lack of sufficient literature regarding the treatment of YS survivors. Due to this poor evidence-base from which to adapt treatments from, this causes a ‘one-size-fits-all’ approach.

It’s almost a wee bit more prescriptive as you get older and then younger people depending what their specific challenges are, you sometimes have to look outside the box and say there’s no evidence base (HCP 9982, L83, age late 40s).

Funding is needed to hire specialised staff, training and equipment to help aid the stroke pathway. Notably, many participants detailed a lack of a dedicated on-site psychologist or mental health specialist within their stroke team. As stated earlier, there is a huge emotional/psychological impact after onset of stroke for both survivors and loved ones, and with no designated support in this area within a lot of stroke teams, many participants found themselves unable to effectively signpost those in need.

We don’t have a specific funding for that (psychological support), so we’re just trying to do what we can with the funding that we have and that can be challenging (HCP 3931, L154, age early 30s).

## Discussion

This qualitative study has explored the attitudes of key stakeholders (YS survivors/caregivers and HCPs) on the impact of younger stroke across five countries. While previous research has explored the poststroke impact and unmet needs of younger stroke, these findings are often expressed using stakeholder data separately.^16^,^19^ The following study is the first to systematically collect and compare the perspectives of three YS stakeholder groups in parallel, revealing both consensus (eg, the psychosocial impact) and dissonance (eg, perceptions on diagnosis speed) in their findings.

It is crucial to understand the complexities apparent among this younger cohort as they have experienced a ‘biographical disruption’, a concept describing how the sudden onset of an injury or illness can disrupt one’s sense of self as well as their thoughts, relationships and future planning.^32 33^ YS survivors are typically in a stage of life where they are engaging in one or several domains that can be disrupted including self-discovery, career development, establishing financial security, starting or maintaining relationships and caring for dependents.^10 11 22^ It is vital that the impact of these disruptions be understood, with the thematic analysis of the interview data showcasing the vast range of poststroke impacts experienced by YS survivors and caregivers, and some of the unmet needs that may arise thereafter.

Exploration into the most impactful domains of young poststroke life revealed that they were similar and shared across all participant groups, with particular focus placed on loss of employment, reliable income and independence, in addition to the overwhelming detrimental effects of YS onset on psychosocial health. Exploring these domains further, it is important to highlight the shock that accompanies these poststroke impacts and their onset during a younger stage of life. For example, careers and general income are often disrupted, but this can subsequently derail long-term career trajectories and long-term financial goals such as pension contributions and child education funds. So while stroke onset can impact on these domains, it is important to also raise that these can lead to lifelong economic insecurities in the years and decades that follow. Consistent with existing research,^22^ this study supports such findings with extensive qualitative evidence that adds greater depth of understanding to age-specific impacts, while also attributing many of these impacts to the caregivers of YS survivors. It was common for YS survivors to be dissatisfied with their care in-hospital due to the ‘one-size-fits-all’ style of treatment they received reminiscent of existing literature.^34 35^ This is supported by HCP responses, which detailed an overall lack of evidence-base with regards to the treatment of YS survivors. Despite being identified in existing research,^36^ this study demonstrates that this lack of evidence base is still an issue in younger stroke healthcare.

Many patients desired more comprehensible, age-specific information and signposting to be provided, particularly in areas involving access to psychosocial and financial support in addition to how one can adjust to various aspects of life after a stroke. Information provision was reportedly more accessible in a community setting, but for many YS survivors and caregivers this was too late. This was particularly prominent with regards to young survivor and caregiver psychological and social support, which many healthcare services were unable to provide. Similarly, follow-up postdischarge was often disparate, unhelpful or non-existent. This may be particularly impactful among YS survivors and their caregivers as they tend to live with the effects of stroke for longer postdischarge.^8^ Despite these trends, many YS survivors accounted on the positive outcomes in their lives poststroke including a calmer lifestyle and a developing sense of community. These views were shared by some caregivers, but these participants often had a more difficult time expressing these positive outcomes, possibly as a result of minimal support in their role.^6^ Despite the low numbers of caregivers in this study, a number of caring-specific impacts were raised, such as the changing relationship between YS survivors and caregivers and the impact stroke can have on caregiver independence and time spent working and caring. All of these unique insights provided by caregivers of this study demonstrate evidence that the impact of younger stroke goes beyond just the survivor.

YS survivors and caregivers often felt like an invisible sub-population within the general stroke population, which was often exemplified when discovering they were often the youngest attendees at peer support groups by a significant margin, leading them to feel more isolated. YS survivor/caregiver support groups are also reportedly less readily available compared to their typically aged counterparts, which could lead to further isolating feelings. Beyond treatment and support groups, many YS survivors wished for more recognition from healthcare and wider society with regards to their stroke. Factors including delayed or missed diagnoses and public misconceptions regarding stroke as a condition of the elderly seem to exacerbate the invisible impact of younger stroke further. Unseen poststroke impairments were shown to be inefficiently treated or untreated in many YS survivors of this study. Fatigue, cognitive impairments, poor psychosocial health, inhibited job prospects and shifts in personality or temperament were all commonly addressed as invisible. Many of these factors, including some cognitive impairments and changes in personality and temperament, were often unregistered by YS survivors, instead being reported by HCPs and YS caregivers respectively. Additionally, many of these YS impairments and poststroke impacts compound on one another, with HCPs highlighting that cognitive impairments may cause disrupted occupations, and then YS survivors raising that this can then lead to increased levels of financial stress. This sequence illustrates the interconnections between different stakeholder interviews, while also showcasing where support needs to be focused in order to mitigate the poststroke impact among young survivors. Accordingly, the following study captures some of the dynamic interconnections between themes (ie, how changes in personality, temperament and mood or familial and romantic relationships can be invisible), leading to an in-depth, multifaceted understanding of the impact of younger stroke.

### Strengths and limitations

With 36 participant interviews across three stakeholder groups, this study adds a rich and in-depth account of the impact of younger stroke. Varying accounts inspired by the experience of YS caregivers and HCPs provide invaluable information into core perceptions surrounding the topic of younger stroke. First-person accounts on various aspects of poststroke life among young survivors help to support and inform findings from existing literature, including lesser visible factors. However, these findings should be considered in light of some methodological issues. Like other qualitative stroke research,^37^ YS caregiver recruitment proved challenging, which may be limiting as the impact of younger stroke may be more pronounced in those who could not participate in this study. Due to the lower levels of YS caregiver engagement in this study, it is likely that data saturation was not reached^24^ for this cohort and there may be more poststroke impacts to explore from this perspective. Despite these lower numbers, there was still commonality within participant responses which allowed some conclusions to be drawn. It is also worth noting that the gender, language and geographical distribution of participant demographics leans towards English-speaking participants (100.00%), female participants (66.66%) and participants recruited from the UK (69.44%). As such, these limitations may shape or constrain the findings of this study so that they may not be as transferable to wider international contexts or capture as many impacts found among other YS cohorts (ie, such as male survivors). Similarly, the recruitment strategy for this study used infographics written in English on social media sites and charity mailing lists, meaning that some findings may not highlight impacts found among those in different non-English-speaking healthcare/social welfare systems or those with lower levels of digital literacy or social media engagement. Finally, participants were not screened for stroke severity. While recruited YS survivors exhibited a range of cognitive/communicative difficulties during interviews, the extent or severity of their impairments were unknown.

### Implications for future research and clinical practice

As YS incidence and prevalence increases,^38 39^ the need for a deeper understanding of this subpopulation also increases. As such, this study recommends that future research investigate aspects of poststroke life among YS survivors compared with that of typically aged survivors utilising a similar multistakeholder approach. Research suggests that caregivers of older stroke survivors often feel unprepared and emotionally distressed when adopting their caregiving role,^40^ but many caregivers of YS survivors are often a partner, spouse or parent who were not expecting to have this responsibility at this stage of life.^6^ Accordingly, investigations should be conducted comparing and aiding the preparedness of caregivers for young/old stroke, especially given the lack of data saturation for this cohort in this study. Regarding life stages of stroke survivors, HCPs highlighted that ages and life stages may not align (eg, those in their thirties may not be raising a family). When examining poststroke impact, care has to be taken when defining what a ‘YS’ is. While the current study uses the Stroke Association’s definition of 18–65 (ie, working age), the needs on the younger end of this bracket (eg, 18–30) may be drastically different to those in older brackets. Considering the differing age definitions, life stages and stroke severities that can influence one’s ability to engage in recovery and one’s capacity for recovery, future research should investigate the weight of these predictive variables and compare them to that of varying age groups within and beyond a YS cohort. Lastly, investigations into the diverse financial implications of YS onset are limited^36^ and therefore warrant further investigation on the implications of this burden.

## Conclusions

In conclusion, the impact younger stroke exhibits on YS survivors, caregivers and their wider circles will continue unless significant changes occur at a clinical, public and procedural level. Accordingly, changes should be implemented in order to target the four themes of impact. Specialised age-appropriate considerations, information provision and signposting should be conducted for YS survivors and caregivers on discharge and throughout the rehabilitation pathway. Such considerations should also be mindful that age and life stage may not align, especially when looking at ranges from 18 to 65. HCPs should be knowledgeable of relevant occupational and financial supports and use interdisciplinary collaboration with occupational therapists, social workers, clinical psychologists, financial advisors and other relevant disciplines in order to tailor these supports to YS survivors poststroke impairments and circumstances. Routine screening and testing for any invisible poststroke deficits (such as including mild cognitive impairment) should be offered at both predischarge and postdischarge, as well as distinct efforts to establish inclusive community support groups for YS survivors and caregivers. Finally, international healthcare practices should revisit the current stringent age-based, service thresholds and work with survivors and caregivers in order to mitigate the impact of younger stroke.

## Supplementary material

10.1136/bmjopen-2025-114949 online supplemental file 1

10.1136/bmjopen-2025-114949 online supplemental file 2

10.1136/bmjopen-2025-114949 online supplemental file 3

## Data Availability

No data are available.
