# Supplementary material for: Exploring key stakeholder perceptions on the impact of younger stroke: a multinational, qualitative interview study
Source: BMJ Open. 2026 Jun 28;16(6):e114949. doi: 10.1136/bmjopen-2025-114949 (PMC13311581; doi:10.1136/bmjopen-2025-114949)
Supplement: online supplemental file 1 [file bmjopen-16-6-s001.docx]

## Supplementary Material 1. – YS Survivor, Caregiver & HCP Interview Schedules

### Young Stroke Survivor Interview Questions

- What do you personally find the most impactful impairment on you post-stroke?
- What was the hardest part of having a stroke? Is there anything you could do prior to your stroke that you can no longer do or now find difficult? Examples?
- Do you think your memory, attention etc. has been effected? How? Maybe your concentration? Your ability to make decisions? Would you say you had good memory/attention before? Has it deteriorated in any way? How?
- Do you feel all your physical needs were met after stroke? What do you think your physical needs are? Mobility? Communication? Fatigue?
- Do you feel all your cognitive needs were met after stroke? What do you think your cognitive needs are? Your thinking?
- Do you feel all your emotional/psychological needs were met after stroke? Did you feel supported or that you were coping well? What do you think your emotional needs are? Your feelings?
- What do you think the biggest change on your life after stroke is? Maybe a good change? Social life? Finances? Work? Were you working when you had your stroke? What aspect of your stroke meant that you could not continue? Impact on your family?
- What unmet needs do you feel you have that need to be addressed? (physical, financial, occupational, cognitive)
- Family support? Spiritual? Social? What do you think of the term “unseen” when referring to the impact of stroke? Should it be used more in the stroke service? Why/why not? Things that you see that impact your life, that others do not.

*Stroke Carer Interview Questions*

- Did you feel supported in your role as a carer for younger stroke survivors soon after their stroke? What about now? Is there any additional support would have been useful?
- What support did you receive? Were there other carers of young stroke survivors you found had similar opinions? Were you the sole carer?
- What has been the biggest impact on your life as a carer post-stroke?
- What was the hardest part? Mobility, communication of survivor? Time allocation? Holidays? Social? Relationship? Family of those under your care? Good impact?
- What causes the biggest impact on the young stroke survivor’s life?
- What do you think was hardest for them? Physical, cognitive etc.? Good impact?
- What do you think of the term “unseen” when referring to the impact of stroke? Should it be used more in the stroke service? Why/why not? Things that you see that impact your life, that others do not.
- What are some unseen needs you have found in those under your care? Have you noticed unseen needs? This could be not paying attention, difficulty remembering or maybe thinking of the right word in a situation.
- What are some unmet needs you have found in those under your care? Physical, emotional, psychological, cognitive? Are any of these partially unmet?
- What unmet needs do you feel need to be raised, either for yourself, or the person you cared for? (physical, financial, occupational, cognitive) Which are most in need of being raised?

*Stroke Clinician Interview Questions*

- Do you think young stroke survivors have specific needs? If so, what? How do these needs differ to that of the general stroke population? How do you think stroke effects young stroke survivor’s lives specifically? Not stroke in general, in young people. Physical, cognitive, social, familial, spiritual etc.?
- What is the follow-up process on young stroke survivor care? Is it different to that of older patients? Why/why not? What is the average duration of treatment? Appointments? Diagnosis time? Time in hospital? How often are you involved in their overall recovery?
- Of physical, cognitive and psychological recovery, which is the most important in young stroke survivors? Which is the least worked on? Why? In your opinion, what aspects of physical/cognitive/psychological recovery are important for young stroke survivors?
- Is there any intervention or treatment that you particularly use with younger stroke survivors? Why/why not? Have you evidenced better results by offering certain services?
- What do you think of the term “unseen?” Do you think young stroke survivors have any particular needs for when they return home? Immediately after they have left your service? Are they risk assessed or do they undergo some form of assessment? If so, what do you use? What does it entail? Is there a minimum threshold of recovery that has to be met before they return home?
