# Supplementary figures and images for: Exploring key stakeholder perceptions on the impact of younger stroke: a multinational, qualitative interview study
Source: BMJ Open. 2026 Jun 28;16(6):e114949. doi: 10.1136/bmjopen-2025-114949 (PMC13311581; doi:10.1136/bmjopen-2025-114949)

***Supplementary Material 2. – Venn Diagram Illustrating Stakeholder Thematic Contributions***

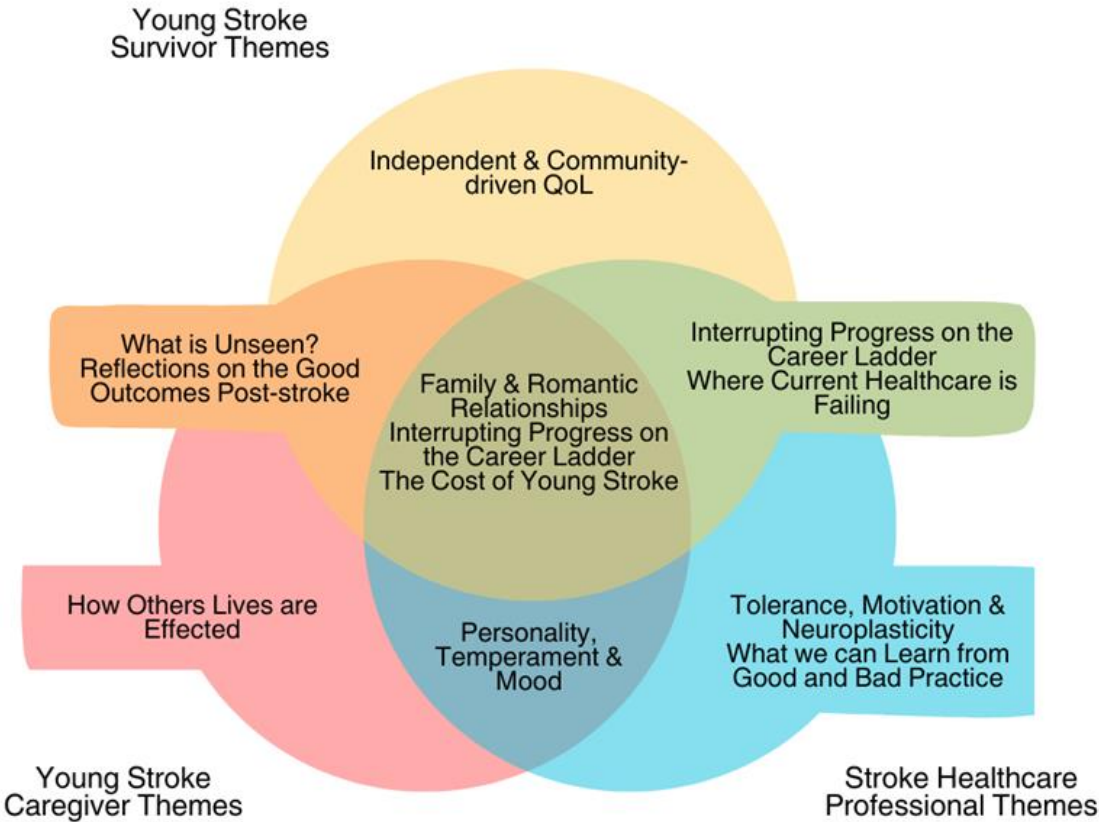

Supplement: online supplemental file 3 [file bmjopen-16-6-s003.pdf]
