# Supplementary material for: Exploring key stakeholder perceptions on the impact of younger stroke: a multinational, qualitative interview study
Source: BMJ Open. 2026 Jun 28;16(6):e114949. doi: 10.1136/bmjopen-2025-114949 (PMC13311581; doi:10.1136/bmjopen-2025-114949)
Supplement: Supplementary file 4 [file bmjopen-2025-114949.reviewer_comments.pdf]

PEER REVIEW HISTORY

BMJ Open publishes all reviews undertaken for accepted manuscripts. Reviewers are asked to complete a checklist review form and are provided with free text boxes to elaborate on their assessment. These free text comments are reproduced below.

ARTICLE DETAILS

Title (Provisional)

Exploring Key Stakeholder Perceptions on the Impact of Younger Stroke – A Multinational, Qualitative Interview Study

Authors

Cleary, Oisin; Davison, Jenny; McBride, Orla; Kennedy, Niamh

VERSION 1 - REVIEW

**Reviewer**	**1**
**Name**	**Mbalinda, Scovia**
**Affiliation**	**Makerere University, Nursing**
**Date**	**18-Feb-2026**
**COI**	**None**

- **Title and abstract**
  The abstract is well-organised and matches the content. You may want to add a specific policy or service implication in the last sentence.
- **Introduction**
  The introduction is short, well-referenced, and makes the problem evident (the growth of young stroke, their special demands, the effects that haven't been handled, the gaps in qualitative work, and the need for a multi-stakeholder perspective).
  -You should make a comparison between your study and already existing studies and what your study adds
  -The goal is evident, but you could want to make it more specific by adding a cross-stakeholder comparison

Methodology

- The qualitative, semi-structured interview approach is adequate and well-articulated; ethical approval and the overall timeline are provided.
- You may need to elaborate: why you chose online 1:1 interviews instead of focus groups or mixed modes?
- The inclusion criteria are clear for each group (survivors, caregivers, HCPs), and it makes sense to focus on clinically diagnosed stroke patients between the ages of 18 and 65.
  It is effectively detailed how to recruit people through charities, professional mailing

lists, and social media. Is there any bias? You may only include those who are digitally literate.
- You only got 5 caregivers, would this give us the depth of analysis to make conclusions
  Steps
- There is a clear description of the interview format, platform (Microsoft Teams), semi-structured approach, and lack of incentives. However, this would be strengthened by the average time per interview, the number of interviews performed by the interviewer, and whether the areas of discussion change during data collection
- Analysis of data: The use of NVivo, thematic analysis, and dual coding for six interviews (two per group) is effectively explained and acceptable. This section can be enhanced by adding details such as how the codes were developed, how the themes were refined, and how you dealt with disagreements among the coders. Also, include a section on reflexivity
- Involvement of Patients and the Public
  It's nice that survivors, caregivers, and healthcare experts contributed draft the interview questions. Make it clear whether the participants looked at the results and helped you make sense of the themes

Results
- **Characteristics of the participants**, most of the demographics are explicitly highlighted – can we also state the imbalance's, like most of the participants are coming from the UK AND HOW CAN THIS AFFECT THE FINDING

- Thematic results: Were there any similarities and contrasts between the health workers and survivors? Go beyond just describing things and add brief linking lines that analyse how factors such as age, life stage, and system design interact to generate specific outcomes. This will make themes seem more interpretative than merely descriptive.
- Discussion
  You could want to add a short conceptual paragraph (for example, using biographical disruption, invisible disability, or family systems) to explain why the effects you talk about are especially important for younger adults.
  The "international" statement should be toned down, as the majority of participants came from the UK, with only 1 each from India and South Africa. Maybe you focused on English-speaking, and maybe we say high-income and middle-income. Most of the people who took part are from the UK. It should also be made clear that these are mostly high-income
- Limitations should be broadened to a small caregiver sample, online/digital bias, and a lack of representation of severe communication/cognitive impairments.
  Consequences and conclusions
- You may make the practice and policy consequences feel more concrete by translating the themes into a short list of particular proposals. For example, structured caregiver information and signposting after release, routine screening for hidden disabilities, age-appropriate vocational and financial coaching, and rethinking stringent age-based service thresholds.
- **Standards for reporting and references**
  If you want to meet BMJ Open's standards, you might briefly mention a qualitative

reporting guideline (such as "reported in line with COREQ") and include the checklist as supplementary materials

**Reviewer**	**2**
**Name**	**Xie, Shiqi**
**Affiliation**	**Chongqing Medical University, Nursing School**
**Date**	**22-Feb-2026**
**COI**	**None**

1.The study explicitly states the challenge of recruiting caregivers (n=5). While acceptable in qualitative research, the authors should further discuss in the "Limitations" section whether this small sample size might have limited data "saturation" or the full exploration of themes for this group, and the potential implications for the study's conclusions.

2.The participant sample shows a high proportion of female survivors (72.73%), and the majority are from the UK (n=25). It is suggested to briefly address in the Discussion or Limitations how the gender and geographical distribution of the sample might affect the generalizability of the findings.

3.The mean age of survivors is 46.77, with the oldest being 72 (but with stroke onset at 58). When discussing impacts specific to "young" individuals (e.g., career disruption, parenting stress), it is recommended that the authors provide a more nuanced analysis of whether need differences exist across age subgroups (e.g., 18-35 vs. 50-65).

4.The presentation of the four main themes is clear, but the Discussion could further elaborate on their interconnections. For example, how "unseen impacts" (e.g., cognitive fatigue) exacerbate "occupational and financial" difficulties, and how this, in turn, affects "relationship and psychosocial" health.

5.The current Conclusion is somewhat general. It is suggested to distill more specific and actionable implications based on the four core themes. For example, regarding "unseen impacts," suggest how to improve clinical screening (especially cognitive screening); regarding "failures in healthcare practices," propose concrete directions for improving follow-up support, information provision, and interdisciplinary collaboration.

VERSION 1 - AUTHOR RESPONSE

Reviewer: 1

Title and abstract

1. The abstract is well-organised and matches the content. You may want to add a specific policy or service implication in the last sentence.

The conclusion of the abstract has been altered to feature service implications stated later in the paper. – "Participants reported a range of post-stroke impacts specific to a younger cohort. These should be considered when developing and engaging in service provision for young stroke survivors to ensure an inclusive, person-led approach that goes beyond 'one-size-fits-all'"

Introduction

2. The introduction is short, well-referenced, and makes the problem evident (the growth of young stroke, their special demands, the effects that haven't been handled, the gaps in qualitative work, and the need for a multi-stakeholder perspective).

Thank you.

3. You should make a comparison between your study and already existing studies and what your study adds

The introduction details what existing studies have demonstrated through individual stakeholder or multistakeholder data collection, and how this study adopts the latter to inform and add to young stroke research. Additional detail has been added in comment 4 that directly relates to making this comparison more clear.

4. The goal is evident, but you could want to make it more specific by adding a cross-stakeholder comparison

In order to make the goal of the study more specific and add a comparison between this study and existing literature (as requested in comment 3), the following has been added to the introduction and the overall study aims. – "This study seeks to establish findings that compound upon those raised in existing literature through its use of multiple perspectives. Accordingly, the following research study aims to explore and compare themes relating to the impact of younger stroke internationally across three key stakeholder groups, in order to better inform future young stroke practice and research."

Methodology

5. The qualitative, semi-structured interview approach is adequate and well-articulated; ethical approval and the overall timeline are provided.

Thank you for your kind words.

6. You may need to elaborate: why you chose online 1:1 interviews instead of focus groups or mixed modes?

   The following has been added to the beginning of the interview procedure section in the methodology. – "One-on-one interviews were conducted online on Microsoft Teams by the first author, following a semi-structured approach for each stakeholder group. This format was chosen to accommodate differing participant schedules and time zones, as well as supporting any potential communication needs exhibited by YS survivors. One-on-one interviews also encouraged a safe environment for participants to discuss any sensitive topics regarding their stroke with no judgement."

7. The inclusion criteria are clear for each group (survivors, caregivers, HCPs), and it makes sense to focus on clinically diagnosed stroke patients between the ages of 18 and 65.

Thank you for your comment.

8. It is effectively detailed how to recruit people through charities, professional mailing lists, and social media. Is there any bias? You may only include those who are digitally literate.

It has been acknowledged that there may be some bias regarding Participants and Recruitment section. – “As recruitment was conducted through these means, there may be potential bias, as all YS survivors and caregivers would need to be digitally literate and engaging with stroke charities or social media groups to access recruitment information.”

9. You only got 5 caregivers, would this give us the depth of analysis to make conclusions

It has been made more clear in the limitations of the study how the low numbers of caregivers may influence the analysis of this particular cohort. – “Like other qualitative stroke research (Leslie et al., 2019), YS caregiver recruitment proved challenging, which may be limiting as the impact of younger stroke may be more pronounced in those who could not participate in this study. Due to the lower levels of YS caregiver engagement in this study, it is likely that data saturation was not reached (Braun & Clarke, 2023) for this cohort and there may be more post-stroke impacts to explore from this perspective. Despite these lower numbers, there was still commonality within participant responses which allowed some conclusions to be drawn.”

Steps

10. There is a clear description of the interview format, platform (Microsoft Teams), semi-structured approach, and lack of incentives. However, this would be strengthened by the average time per interview, the number of interviews performed by the interviewer, and whether the areas of discussion change during data collection

Detail on number of interviews is highlighted at the beginning of the results section (n=36). The following details have been added to the interview procedure in order to capture who the interviewer was and how areas of discussion was led by participant responses. Average interview time has been moved from the results section to the procedure – “1-on-1 interviews were conducted online on Microsoft Teams by the first author… All participant interviews followed a consistent interview schedule for each stakeholder group (see Supplementary Material 1.), with variation emerging in participant responses… Interview duration was a mean average of 49.51 (SD=17.48) minutes.”

11. Analysis of data: The use of NVivo, thematic analysis, and dual coding for six interviews (two per group) is effectively explained and acceptable. This section can be enhanced by adding details such as how the codes were developed, how the themes were refined, and how you dealt with disagreements among the coders. Also, include a section on reflexivity

Several points have been added to the data analysis section to account for how codes were developed, how themes were refined, how disagreements were tackled (if they were to emerge) and researcher reflexivity. – “Codes were developed using short phrases that described the theme/subtheme being discussed by participants (e.g., “occupational impact”)… Both authors identified themes separately across six participant interviews (two for each stakeholder group), then compared coding to ensure rigour, refinement and consensus of emerging themes. Interpretation of participant interviews falls on author reflexivity, and so it is important to highlight that the first author was an early career researcher new to qualitative research practices whereas the second author had several years more experience as a qualitative researcher. Though no conflicts arose during this process, the third author was ready to mediate this process.”

Involvement of Patients and the Public

12. It's nice that survivors, caregivers, and healthcare experts contributed draft the interview questions. Make it clear whether the participants looked at the results and helped you make sense of the themes

It is made clearer that survivors, caregivers and healthcare professionals were not involved during "the data analysis, reporting or dissemination of the research".

Results

13. Characteristics of the participants, most of the demographics are explicitly highlighted – can we also state the imbalance's, like most of the participants are coming from the UK AND HOW CAN THIS AFFECT THE FINDING

It is made clearer that majority of participants are from the UK. – "The majority (69.44%) of participants were recruited from the UK (n=25), with other international participation involving Canada (n=5), the USA (n=4), India (n=1) and South Africa (n=1)."

This is later raised as a limitation of the study in the discussion section. – "It is also worth noting that the gender, language and geographical distribution of participant demographics leans towards English-speaking participants (100.00%), female participants (66.66%) and participants recruited from the UK (69.44%). As such, the findings of this study may not be as transferable to wider international contexts or capture as many impacts found among young male stroke survivors."

14. Thematic results: Were there any similarities and contrasts between the health workers and survivors? Go beyond just describing things and add brief linking lines that analyse how factors such as age, life stage, and system design interact to generate specific outcomes. This will make themes seem more interpretative than merely descriptive.

Similarities and contrasts between health workers and survivors (and caregivers) are highlighted in the discussion section. Data collection featured separate interview schedules for each participant group and so the results currently reflect this. The discussion is where these comparisons and how links between most impactful domains, one-size-fits-all treatment plans, information provision etc. are explored. For example. – "Information provision was reportedly more accessible in a community setting, but for many this was too late. This was particularly prominent with regards to young survivor and caregiver psychological and social support, which many healthcare services were unable to provide."

Discussion

15. You could want to add a short conceptual paragraph (for example, using biographical disruption, invisible disability, or family systems) to explain why the effects you talk about are especially important for younger adults.

A short paragraph and accompanying references have been added to explain biographical disruption and how it can be used to explain why the post-stroke impacts raised are of particular importance to YS survivors. – "It is crucial to understand the complexities apparent among this younger cohort has they have experienced a 'biographical disruption', a concept describing how the sudden onset of an injury or illness can disrupt one's sense of self as well as their thoughts, relationships and future planning (Bury 1982; Pranka, 2018). YS survivors are typically in a stage of life where they are engaging in one or several domains that can be disrupted including self-discovery, career development, establishing financial security, starting or maintaining relationships and caring for dependents (Harris Walker et al., 2021; Martinsen et

al., 2012; Morris 2011). It is vital that the impact these disruptions have be understood, with the thematic analysis of the interview data…"

16. The "international" statement should be toned down, as the majority of participants came from the UK, with only 1 each from India and South Africa. Maybe you focused on English-speaking, and maybe we say high-income and middle-income. Most of the people who took part are from the UK. It should also be made clear that these are mostly high-income

We appreciate this comment and have reflected this in our revised draft. Examples include - "the impact of younger stroke across five countries (instead of an international context)"… "A Multinational, Qualitative Interview Study (formerly international)". This is also made more explicit as a limitation in line with comment 17.

17. Limitations should be broadened to a small caregiver sample, online/digital bias, and a lack of representation of severe communication/cognitive impairments.

Limitations have been updated to clearly state the UK recruited and English-speaking majority. Furthermore, gender distribution has also been raised as a potential limitation in line with other reviewer comments. The small caregiver sample is already stated as a limitation of the study backed by existing literature. Severe communication/cognitive impairments are raised at the end of the limitations, particularly how even though participants exhibited these impairments, the severity is unknown. Income was a core element explored in the financial impact of young stroke, with participants coming from a wide range of low, medium and high income backgrounds. Similarly, digital literacy has been mentioned in the Participants and Recruitment section in line with review comment 8. – "It is also worth noting that the gender, language and geographical distribution of participant demographics leans towards English-speaking participants (100.00%), female participants (66.66%) and participants recruited from the UK (69.44%). As such, the findings of this study may not be as transferable to wider international contexts or capture as many impacts found among young male stroke survivors. Finally, participants were not screened for stroke severity. While recruited YS survivors exhibited a range of cognitive/communicative difficulties during interviews, the extent or severity of their impairments were unknown."

Consequences and conclusions

18. You may make the practice and policy consequences feel more concrete by translating the themes into a short list of particular proposals. For example, structured caregiver information and signposting after release, routine screening for hidden disabilities, age-appropriate vocational and financial coaching, and rethinking stringent age-based service thresholds.

The conclusion has been rewritten to offer more specific suggestions for practice and policy in line with the four identified themes. - "In conclusion, the impact younger stroke exhibits on YS survivors, caregivers and their wider circles will continue unless significant changes occur at a clinical, public and procedural level. Accordingly, changes should be implemented in order to target the four themes of impact. Specialized age-appropriate considerations, information provision and signposting should be conducted for YS survivors and caregivers upon discharge and throughout the rehabilitation pathway. HCPs should be knowledgeable of relevant occupational and financial supports and utilise interdisciplinary collaboration to tailor these supports to YS survivors post-stroke impairments and circumstances. Routine screening and

testing for any invisible post-stroke deficits (such as including mild cognitive impairment) should be offered at both pre- and post-discharge, as well as distinct efforts to establish inclusive community support groups for YS survivors and caregivers. Finally, international healthcare practices should revisit the current stringent age-based, service thresholds and work with survivors and caregivers in order to mitigate the impact of younger stroke."

Standards for reporting and references

19. If you want to meet BMJ Open's standards, you might briefly mention a qualitative reporting guideline (such as "reported in line with COREQ") and include the checklist as supplementary materials

In line with the editors comments, the SRQR checklist has been added as supplementary material 3. Readers are signposted towards this in The scoring checklist is included in supplementary materials. We have also addressed COREQ in methods

Reviewer: 2

1. The study explicitly states the challenge of recruiting caregivers (n=5). While acceptable in qualitative research, the authors should further discuss in the "Limitations" section whether this small sample size might have limited data "saturation" or the full exploration of themes for this group, and the potential implications for the study's conclusions.

A sentence has been added to the limitations further exploring the lack of data saturation for this cohort and how there may be more to explore in this area. – "Due to the lower levels of YS caregiver engagement in this study, it is likely that data saturation was not reached (Braun & Clarke, 2023) for this cohort and there may be more post-stroke impacts to explore from this perspective."

2. The participant sample shows a high proportion of female survivors (72.73%), and the majority are from the UK (n=25). It is suggested to briefly address in the Discussion or Limitations how the gender and geographical distribution of the sample might affect the generalizability of the findings.

In line with the other reviewers comments, limitations have been updated to clearly state high-proportions of English-speaking participants who were female and recruited from the UK. – "It is also worth noting that the gender, language and geographical distribution of participant demographics leans towards English-speaking participants (100.00%), female participants (66.66%) and participants recruited from the UK (69.44%). As such, the findings of this study may not be as transferable to wider international contexts or capture as many impacts found among young male stroke survivors."

3. The mean age of survivors is 46.77, with the oldest being 72 (but with stroke onset at 58). When discussing impacts specific to "young" individuals (e.g., career disruption, parenting stress), it is recommended that the authors provide a more nuanced analysis of whether need differences exist across age subgroups (e.g., 18-35 vs. 50-65).

There is also a quote addressing some of this nuance from an occupational/financial perspective in the Results - The Cost of Young Stroke. This nuance has been made more clear in the analysis – "Additional costs when making adjustments to living areas, early pensions/retirements, excessive sick pay, dipping into savings and the subsequent financial

stress of all these areas were all demonstrated, causing a great need for increased financial aid post-stroke amid younger individuals. Notably, many of these adjustments may already exist or not be necessary in typically aged stroke survivors or in older survivors within the 18-65 bracket (e.g., those aged 55-65) who may already be retired or near retirement, providing an extra layer of financial assurance unavailable to younger survivors and caregivers, who may have more costs to consider. "More of my people who are older and have had strokes are more prepared to retire. Umm, my people, who are, you know, like say 27-32, are not ready to retire" (HCP 0782, L66, aged 31)."

Further discussion has also been added to the Implications for Future Research & Clinical Practice section of the Discussion detailing some more nuance to defining a younger stroke, and offering future research suggestions to breaking down age brackets into potentially more appropriate subgroups when examining young stroke impact. – "When examining post-stroke impact, care has to be taken when defining what a 'young stroke' is. While the current study use the Stroke Association's definition of 18-65 (i.e., working age), the needs on the younger end of this bracket (e.g., 18-30) may be drastically different to those in older brackets. Considering the differing age definitions, life stages and stroke severities that can influence one's ability to engage in recovery and one's capacity for recovery, future research should investigate the weight of these predictive variables and compare them to that of varying age groups within and beyond a young stroke cohort." –

4. The presentation of the four main themes is clear, but the Discussion could further elaborate on their interconnections. For example, how "unseen impacts" (e.g., cognitive fatigue) exacerbate "occupational and financial" difficulties, and how this, in turn, affects "relationship and psychosocial" health.

More detail has been added to the discussion to elaborate on the interconnections between stakeholder interviews, as well as showing how the themes can relate to one another. It just so happens the example the reviewer provided was one that emerged in the data set. – "Fatigue, cognitive impairments, poor psychosocial health, inhibited job prospects and shifts in personality or temperament were all commonly addressed as invisible. Many of these factors, including some cognitive impairments and changes in personality and temperament were often unregistered by YS survivors, instead being reported by HCPs and YS caregivers respectively. Additionally, many of these YS impairments and post-stroke impacts compound upon one another, with HCPs highlighting that cognitive impairments may cause disrupted occupations, and then YS survivors raising that this can then lead to increased levels of financial stress. This sequence illustrates the interconnections between different stakeholder interviews, whilst also showcasing where support needs to be focused in order to mitigate the post-stroke impact among young survivors."

5. The current Conclusion is somewhat general. It is suggested to distill more specific and actionable implications based on the four core themes. For example, regarding "unseen impacts," suggest how to improve clinical screening (especially cognitive screening); regarding "failures in healthcare practices," propose concrete directions for improving follow-up support, information provision, and interdisciplinary collaboration.

In line with this comment and the other reviewers comment, the conclusion has been rewritten to offer more specific and actionable implications based on the four core themes. – "In conclusion, the impact younger stroke exhibits on YS survivors, caregivers and their wider circles will continue unless significant changes occur at a clinical, public and procedural level. Accordingly, changes should be implemented in order to target the four themes of impact.

Specialized age-appropriate considerations, information provision and signposting should be conducted for YS survivors and caregivers upon discharge and throughout the rehabilitation pathway. HCPs should be knowledgeable of relevant occupational and financial supports and utilise interdisciplinary collaboration to tailor these supports to YS survivors post-stroke impairments and circumstances. Routine screening and testing for any invisible post-stroke deficits (such as including mild cognitive impairment) should be offered at both pre- and post-discharge, as well as distinct efforts to establish inclusive community support groups for YS survivors and caregivers. Finally, international healthcare practices should revisit the current stringent age-based, service thresholds and work with survivors and caregivers in order to mitigate the impact of younger stroke."

Reviewer: 1

If you have selected 'Yes' above, please provide details of any competing interests.: Not applicable

Reviewer: 2

If you have selected 'Yes' above, please provide details of any competing interests.: Not applicable

---

VERSION 2 - REVIEW

**Reviewer**	**1**
**Name**	**Mbalinda, Scovia**
**Affiliation**	**Makerere University, Nursing**
**Date**	**26-Mar-2026**
**COI**	

Manuscript ID: bmjopen-2025-114949.R1

Title: Exploring Key Stakeholder Perceptions on the Impact of Younger Stroke – A Multinational, Qualitative Interview Study

I write in response to the revised submission of the above-referenced manuscript. Having carefully reviewed the authors' point-by-point response letter and the revised manuscript, I am pleased to confirm that all comments raised by the Editor and both Reviewers have been thoroughly and satisfactorily addressed.

Compared to the initial submission, the amended manuscript is a major improvement. All of the editor's and both reviewers' criticisms have been satisfactorily addressed by the writers' comprehensive, intellectually honest, and suitably deep responses. A conceptually grounded discussion, significant cross-stakeholder comparisons, a fully revised, actionable conclusion that directly addresses the study's four core themes, increased methodological transparency

regarding reflexivity, code development, and interview rationale, and more robust and expansive limitations are among the major improvements.

Substantive revisions are also evident in the restructuring of the title and abstract, the addition of an SRQR checklist, and the careful strengthening of the discussion, limitations, and conclusion sections. These enhancements have brought the reporting in line with the standards expected for qualitative research in BMJ Open.

The manuscript is well-positioned to contribute meaningfully to the young stroke literature, particularly given its novel multi-stakeholder approach and its focus on a population that remains underrepresented in qualitative research.

My recommendation is: ACCEPT FOR PUBLICATION. No further revisions are required.

**Reviewer**	**2**
**Name**	**Xie, Shiqi**
**Affiliation**	**Chongqing Medical University, Nursing School**
**Date**	**20-Mar-2026**
**COI**	

1.The study clearly acknowledges the difficulty in recruiting caregivers (n=5) and the limitations regarding sample gender, language, and geographical skew (predominantly UK-based, English-speaking, female). It is recommended to further elaborate in the “Discussion – Limitations” section on how these limitations specifically affect the transferability of the findings. For instance, the experiences and (online) support accessible to English-speaking participants proficient in using social media and charity resources may not represent those with lower digital literacy or in different healthcare/social welfare systems. Additionally, while the small caregiver sample may not have reached data saturation, the consistency of the emerging themes (e.g., feeling abandoned, immense burden) could be highlighted as identifying key areas for future research.

2.Theme 3 (Out of Sight, Out of Mind): This is a key contribution. It is recommended to more explicitly link the sub-points of “atypical symptoms causing delayed diagnosis” and “public misconception of stroke as an elderly condition” in the Discussion. One could argue that the prevalent misconception may lead patients, families, and even first-contact doctors to dismiss stroke symptoms in the young, exacerbating the “invisible” problem. This strengthens the internal logic of the theme and highlights the need for public education.

Theme 2 (Financial Worries): The study mentions loss of income, out-of-pocket payments, and early retirement. The discussion could more concretely tie this financial shock to the “young” life stage. For example, it’s not just about current income but potentially derailing career trajectories, affecting long-term pension accrual, children’s education funds, etc.,

creating lifecycle economic insecurity fundamentally different from older survivors near or in retirement.

Reflections on Good Outcomes: Theme 3.3 is a strength. Briefly discuss in the Discussion why caregivers found it harder to identify positive outcomes than survivors. Could this be related to the often-overlooked caregiver role, with their distress being more “invisible” and lacking targeted support? This would reinforce the call for tailored psychosocial support for caregivers.

3.The discussion engages with relevant literature. It is recommended to more succinctly articulate the unique value added by this study compared to existing literature, perhaps at the end of the Introduction or beginning of the Discussion. Emphasize that while prior studies have explored the needs of young stroke survivors or caregivers separately, this study is the first to systematically collect and compare the perspectives of survivors, caregivers, and HCPs in parallel, revealing both consensus (e.g., on profound financial and psychosocial impact) and dissonance (e.g., perceptions of diagnosis speed). This provides a stronger foundation for designing coordinated, comprehensive support services.

4.The recommendations in the Conclusion (e.g., age-appropriate services, routine screening for invisible deficits, inclusive support groups) are appropriate. Consider adding a more specific recommendation on “enhancing interdisciplinary team collaboration”, explicitly including occupational therapists, social workers, clinical psychologists, and financial advisors to address the interconnected, multi-dimensional challenges revealed (e.g., cognitive impairments affecting employment, leading to financial crisis). This makes the implications more actionable.

VERSION 2 - AUTHOR RESPONSE

Reviewer: 1

I write in response to the revised submission of the above-referenced manuscript. Having carefully reviewed the authors' point-by-point response letter and the revised manuscript, I am pleased to confirm that all comments raised by the Editor and both Reviewers have been thoroughly and satisfactorily addressed.

Compared to the initial submission, the amended manuscript is a major improvement. All of the editor's and both reviewers' criticisms have been satisfactorily addressed by the writers' comprehensive, intellectually honest, and suitably deep responses. A conceptually grounded discussion, significant cross-stakeholder comparisons, a fully revised, actionable conclusion that directly addresses the study's four core themes, increased methodological transparency regarding reflexivity, code development, and interview rationale, and more robust and expansive limitations are among the major improvements.

Substantive revisions are also evident in the restructuring of the title and abstract, the addition of an SRQR checklist, and the careful strengthening of the discussion, limitations,

and conclusion sections. These enhancements have brought the reporting in line with the standards expected for qualitative research in BMJ Open.

The manuscript is well-positioned to contribute meaningfully to the young stroke literature, particularly given its novel multi-stakeholder approach and its focus on a population that remains underrepresented in qualitative research.

My recommendation is: ACCEPT FOR PUBLICATION. No further revisions are required.

Thank you for your very kind words and for your favourable recommendation for publication.

Reviewer: 2

1) The study clearly acknowledges the difficulty in recruiting caregivers (n=5) and the limitations regarding sample gender, language, and geographical skew (predominantly UK-based, English-speaking, female).

Thank you.

   a) It is recommended to further elaborate in the "Discussion – Limitations" section on how these limitations specifically affect the transferability of the findings. For instance, the experiences and (online) support accessible to English-speaking participants proficient in using social media and charity resources may not represent those with lower digital literacy or in different healthcare/social welfare systems.

Additional detail has been added to the Discussion – Limitations providing another example of how the results may not be transferable among those with lower digital literacy or in different healthcare systems. – "Similarly, the recruitment strategy for this study utilised infographics written in English on social media sites and charity mailing lists, meaning that some findings may not highlight impacts found among those in different non-English-speaking healthcare/social welfare systems or those with lower levels of digital literacy or social media engagement."

   b) Additionally, while the small caregiver sample may not have reached data saturation, the consistency of the emerging themes (e.g., feeling abandoned, immense burden) could be highlighted as identifying key areas for future research.

This recommendation was in the Discussion – Implications for Future Research & Clinical Practice Section, but has been tied in more clearly with the small caregiver sample to strengthen the recommendation. – "Accordingly, investigations should be conducted comparing and aiding the preparedness of caregivers for young/old stroke, especially given the lack of data saturation for this cohort in this study"

2) Theme 3 (Out of Sight, Out of Mind): This is a key contribution.
   a) It is recommended to more explicitly link the sub-points of "atypical symptoms causing delayed diagnosis" and "public misconception of stroke as an elderly

condition" in the Discussion. One could argue that the prevalent misconception may lead patients, families, and even first-contact doctors to dismiss stroke symptoms in the young, exacerbating the "invisible" problem. This strengthens the internal logic of the theme and highlights the need for public education.

Additional discussions have been added to highlight the invisible elements of stroke and how these can influence the invisible impact of young stroke overall – "Beyond treatment and support groups, many YS survivors wished for more recognition from healthcare and wider society with regards to their stroke. Factors including delayed or missed diagnoses and public misconceptions regarding stroke as a condition of the elderly seem to exacerbate the invisible impact of younger stroke further."

b) Theme 2 (Financial Worries): The study mentions loss of income, out-of-pocket payments, and early retirement. The discussion could more concretely tie this financial shock to the "young" life stage. For example, it's not just about current income but potentially derailing career trajectories, affecting long-term pension accrual, children's education funds, etc., creating lifecycle economic insecurity fundamentally different from older survivors near or in retirement.

Three sentences have been added to the discussion to reflect the long-term financial impact as a result of experiencing a stroke at a young age. – "Exploring these domains further, it is important to highlight the shock that accompanies these post-stroke impacts and their onset during a younger stage of life. For example, careers and general income are often disrupted, but this can subsequently derail long-term career trajectories and long-term financial goals such as pension contributions and child education funds. So while stroke onset can impact upon these domains, it is important to also raise that these can lead to lifelong economic insecurities in the years and decades that follow. "

c) Reflections on Good Outcomes: Theme 3.3 is a strength. Briefly discuss in the Discussion why caregivers found it harder to identify positive outcomes than survivors. Could this be related to the often-overlooked caregiver role, with their distress being more "invisible" and lacking targeted support? This would reinforce the call for tailored psychosocial support for caregivers.

A brief discussion has been added exploring why young stroke caregivers may have difficulty expressing positive outcomes post-stroke. – "Similarly, the recruitment strategy for this study utilised infographics written in English on social media sites and charity mailing lists, meaning that some findings may not highlight impacts found among those in different non-English-speaking healthcare/social welfare systems or those with lower levels of digital literacy or social media engagement."

This then ties in better to the aforementioned changes made to the Discussion – Implications for Future Research & Clinical Practice Section as highlighted in Reviewer Comment 1.A.) – "Accordingly, investigations should be conducted comparing and aiding the preparedness of

caregivers for young/old stroke, especially given the lack of data saturation for this cohort in this study."

3) The discussion engages with relevant literature. It is recommended to more succinctly articulate the unique value added by this study compared to existing literature, perhaps at the end of the Introduction or beginning of the Discussion. Emphasize that while prior studies have explored the needs of young stroke survivors or caregivers separately, this study is the first to systematically collect and compare the perspectives of survivors, caregivers, and HCPs in parallel, revealing both consensus (e.g., on profound financial and psychosocial impact) and dissonance (e.g., perceptions of diagnosis speed). This provides a stronger foundation for designing coordinated, comprehensive support services.

The following has been added to the beginning of the Discussion section in line with recommendations. – "This qualitative study is the first to has explored the attitudes of key stakeholders (YS survivors/caregivers and HCPs) on the impact of younger stroke across five countries. While previous research has explored the post-stroke impact and unmet needs of younger stroke, these findings are often expressed using stakeholder data separately16-19. The following study is the first to systematically collect and compare the perspectives of three YS stakeholder groups in parallel, revealing both consensus (e.g., the psychosocial impact) and dissonance (e.g., perceptions on diagnosis speed) in their findings."

4) The recommendations in the Conclusion (e.g., age-appropriate services, routine screening for invisible deficits, inclusive support groups) are appropriate. Consider adding a more specific recommendation on "enhancing interdisciplinary team collaboration", explicitly including occupational therapists, social workers, clinical psychologists, and financial advisors to address the interconnected, multi-dimensional challenges revealed (e.g., cognitive impairments affecting employment, leading to financial crisis). This makes the implications more actionable.

An additional line has been added to the conclusion to explicitly include these professionals in order to make the conclusion more actionable. – "HCPs should be knowledgeable of relevant occupational and financial supports and utilise interdisciplinary collaboration with occupational therapists, social workers, clinical psychologists, financial advisors and other relevant disciplines in order to tailor these supports to YS survivors post-stroke impairments and circumstances."

Reviewer: 1

If you have selected 'Yes' above, please provide details of any competing interests.: Not applicable

Reviewer: 2

If you have selected 'Yes' above, please provide details of any competing interests.: Not applicable

VERSION 3 - REVIEW

**Reviewer**	**2**
**Name**	**Xie, Shiqi**
**Affiliation**	**Chongqing Medical University, Nursing School**
**Date**	**05-May-2026**
**COI**	

1.The authors candidly note limitations such as difficulty recruiting caregivers and the demographic composition (English-speaking, female-dominated) potentially limiting generalizability. These are important reflections but could be discussed more critically regarding how these limitations specifically shaped or potentially constrained the findings.

2.The four themes are robust and well-supported. The use of "biographical disruption" theory provides a good framework. However, the dynamic interconnections between themes and how they collectively constitute the "impact" could be presented more clearly.

3.The study wisely notes that "age and life stage may not align" and that needs may differ vastly between an 18-year-old and a 60-year-old. This is a key insight that could inform more nuanced conclusions and recommendations.

4.While data saturation for caregivers was likely not reached, the paper could more actively discuss the profound, potentially unique insights from the limited caregiver data that was collected.

VERSION 3 - AUTHOR RESPONSE

Reviewer: 2

1. The authors candidly note limitations such as difficulty recruiting caregivers and the demographic composition (English-speaking, female-dominated) potentially limiting generalizability. These are important reflections but could be discussed more critically regarding how these limitations specifically shaped or potentially constrained the findings.

How these limitations may shape or constrain the findings of this study have been made more clear in the Strengths and Limitations section of the discussion – "It is also worth noting that the gender, language and geographical distribution of participant demographics leans towards English-speaking participants (100.00%), female participants (66.66%) and participants recruited from the UK (69.44%). As such, these limitations may shape or constrain the findings of this study so that they may not be as transferable to wider international contexts or capture as many impacts found among other young stroke cohorts

(i.e., such as male survivors)young male stroke survivors. Similarly, the recruitment strategy for this study utilised infographics written in English on social media sites and charity mailing lists, meaning that some findings may not highlight impacts found among those in different non-English-speaking healthcare/social welfare systems or those with lower levels of digital literacy or social media engagement."

2. The four themes are robust and well-supported. The use of "biographical disruption" theory provides a good framework. However, the dynamic interconnections between themes and how they collectively constitute the "impact" could be presented more clearly.

An additional example has been added to the discussion section to better illustrate the interconnectedness of themes and their contribution to the overall impact of younger stroke – "many of these YS impairments and post-stroke impacts compound upon one another, with HCPs highlighting that cognitive impairments may cause disrupted occupations, and then YS survivors raising that this can then lead to increased levels of financial stress. This sequence illustrates the interconnections between different stakeholder interviews, whilst also showcasing where support needs to be focused in order to mitigate the post-stroke impact among young survivors. Accordingly, the following study captures some of the dynamic interconnections between themes (i.e., how changes in personality, temperament & mood or familial and romantic relationships can be invisible), leading to an in-depth, multi-faceted understanding on the impact of younger stroke"

3. The study wisely notes that "age and life stage may not align" and that needs may differ vastly between an 18-year-old and a 60-year-old. This is a key insight that could inform more nuanced conclusions and recommendations.

An additional sentence has been added into the conclusion to reflect this key insight. – "Specialized age-appropriate considerations, information provision and signposting should be conducted for YS survivors and caregivers upon discharge and throughout the rehabilitation pathway. Such considerations should also be mindful that age and life stage may not align, especially when looking at ranges from 18 to 65."

4. While data saturation for caregivers was likely not reached, the paper could more actively discuss the profound, potentially unique insights from the limited caregiver data that was collected.

The following has been added to the discussion in line with reviewer suggestions – "Despite the low numbers of caregivers in this study, a number of caring-specific impacts were raised, such as the changing relationship between YS survivors and caregivers and the impact stroke can have on caregiver independence and time spent working and caring. All of these unique insights provided by caregivers of this study demonstrate evidence that the impact of younger stroke goes beyond just the survivor."
